# TIM1 (HAVCR1) Is Not Essential for Cellular Entry of Either Quasi-enveloped or Naked Hepatitis A Virions

**DOI:** 10.1128/mBio.00969-17

**Published:** 2017-09-05

**Authors:** Anshuman Das, Asuka Hirai-Yuki, Olga González-López, Bethany Rhein, Sven Moller-Tank, Rachel Brouillette, Lucinda Hensley, Ichiro Misumi, William Lovell, John M. Cullen, Jason K. Whitmire, Wendy Maury, Stanley M. Lemon

**Affiliations:** a Lineberger Comprehensive Cancer Center, The University of North Carolina at Chapel Hill, Chapel Hill, North Carolina, USA; b Department of Microbiology, University of Iowa, Iowa City, Iowa, USA; c Department of Population Health & Pathobiology, North Carolina State University College of Veterinary Medicine, Raleigh, North Carolina, USA; d Department of Genetics, The University of North Carolina at Chapel Hill, Chapel Hill, North Carolina, USA; e Department of Microbiology & Immunology, The University of North Carolina at Chapel Hill, Chapel Hill, North Carolina, USA; f Department of Medicine, The University of North Carolina at Chapel Hill, Chapel Hill, North Carolina, USA; Virginia Polytechnic Institute and State University

**Keywords:** hepatitis A virus, hepatovirus, phosphatidylserine, picornavirus, receptor, viral attachment

## Abstract

Receptor molecules play key roles in the cellular entry of picornaviruses, and TIM1 (HAVCR1) is widely accepted to be the receptor for hepatitis A virus (HAV), an unusual, hepatotropic human picornavirus. However, its identification as the hepatovirus receptor predated the discovery that hepatoviruses undergo nonlytic release from infected cells as membrane-cloaked, quasi-enveloped HAV (eHAV) virions that enter cells via a pathway distinct from naked, nonenveloped virions. We thus revisited the role of TIM1 in hepatovirus entry, examining both adherence and infection/replication in cells with clustered regularly interspaced short palindromic repeat (CRISPR)/Cas9-engineered TIM1 knockout. Cell culture-derived, gradient-purified eHAV bound Huh-7.5 human hepatoma cells less efficiently than naked HAV at 4°C, but eliminating TIM1 expression caused no difference in adherence of either form of HAV, nor any impact on infection and replication in these cells. In contrast, TIM1-deficient Vero cells showed a modest reduction in quasi-enveloped eHAV (but not naked HAV) attachment and replication. Thus, TIM1 facilitates quasi-enveloped eHAV entry in Vero cells, most likely by binding phosphatidylserine (PtdSer) residues on the eHAV membrane. Both *Tim1*^*−/−*^* Ifnar1*^*−/−*^ and *Tim4*^*−/−*^* Ifnar1*^*−/−*^ double-knockout mice were susceptible to infection upon intravenous challenge with infected liver homogenate, with fecal HAV shedding and serum alanine aminotransferase (ALT) elevations similar to those in *Ifnar1*^*−/−*^ mice. However, intrahepatic HAV RNA and ALT elevations were modestly reduced in *Tim1*^*−/−*^*Ifnar1*^*−/−*^ mice compared to *Ifnar1*^*−/−*^ mice challenged with a lower titer of gradient-purified HAV or eHAV. We conclude that TIM1 is not an essential hepatovirus entry factor, although its PtdSer-binding activity may contribute to the spread of quasi-enveloped virus and liver injury in mice.

## IMPORTANCE

T cell immunoglobulin and mucin-containing domain protein 1 (TIM1) was reported more than 2 decades ago to be an essential cellular receptor for hepatitis A virus (HAV), a picornavirus in the *Hepatovirus* genus, resulting in its designation as “hepatitis A virus cellular receptor 1” (HAVCR1) by the Human Genome Organization Gene Nomenclature Committee. However, recent studies have shown that HAV exists in nature as both naked, nonenveloped (HAV) virions and membrane-cloaked, quasi-enveloped infectious virus (eHAV), prompting us to revisit the role of TIM1 in viral entry. We show here that TIM1 (HAVCR1) is not an essential cellular receptor for HAV entry into cultured cells or required for viral replication and pathogenesis in permissive strains of mice, although it may facilitate early stages of infection by binding phosphatidylserine on the eHAV surface. This work thus corrects the published record and sets the stage for future efforts to identify specific hepatovirus entry factors.

## INTRODUCTION

Hepatitis A virus (HAV) is a unique, hepatotropic human picornavirus that circulates in blood during acute infection as membrane-cloaked, quasi-enveloped virus (eHAV) but is shed in feces as naked, nonenveloped virions (1). Virus shed in feces is produced largely, if not entirely, within infected hepatocytes (2) and gains access to the gut following its nonlytic release as quasi-enveloped virus across the apical hepatocellular membrane into the biliary track (3). The membranes surrounding the eHAV capsid are removed by the high concentrations of bile acids present within the proximal biliary track, resulting in the shedding of naked virions in feces (3). Low-passage, cell culture-adapted HAV variants such as HM175/p16 (4) replicate in permissive cell cultures without cytopathic effect, with quasi-enveloped eHAV representing the large majority of extracellular virus particles found in supernatant fluids. With respect to their size, buoyant density, and host protein composition, these virions are similar to exosomes, small extracellular vesicles released from cells via the multivesicular body (MVB) pathway (1, 5, 6). The nonlytic release of eHAV from cells is dependent upon ALIX and other proteins associated with endosomal sorting complexes required for transport (ESCRT), and the underlying mechanism is likely to closely mirror the biogenesis of exosomes (1, 5).

Only quasi-enveloped eHAV virions can be detected in plasma or serum from infected humans or experimentally infected chimpanzees (1). The membranes surrounding the capsid in eHAV virions contain no virally encoded proteins (1, 5), a feature that distinguishes quasi-enveloped viruses from conventional enveloped viruses that display viral glycoproteins on their surface (7). The eHAV membrane sequesters the capsid from detection by B cells and protects it from neutralizing antibodies (1), thereby facilitating spread of the virus within the liver. On the other hand, naked virions shed in feces are stable and highly resistant to drying, promoting the capacity of the virus to spread through the environment to naive hosts. This dual lifestyle thus provides HAV with unique advantages for its survival and transmission within susceptible populations. It is not unique to HAV, as hepatitis E virus (HEV), an unrelated hepatotropic, positive-strand RNA virus, is also released from cells and circulates in blood as quasi-enveloped virus but is shed in feces as naked, nonenveloped virions (7).

Although physically distinct, the quasi-enveloped eHAV form of the virus is as infectious as naked virions in cultured cells (1). Little is known about how these two forms of the virus enter cells, although there are clear differences in their entry mechanisms. eHAV entry is slow and sensitive to the lysosomal poison chloroquine. Neutralizing anti-capsid antibodies restrict replication of eHAV when added to cultures as late as 6 h after infection, reflecting neutralization within an endocytic compartment, most likely lysosomes, in which the enveloping membrane has been degraded (1). In contrast, naked HAV enters rapidly, is resistant to chloroquine, and is not subject to postendocytic neutralization. Receptor-capsid interactions are crucial for uncoating and cytoplasmic delivery of the genomes of other picornaviruses (8), but how this relates to the entry of HAV and the quasi-enveloped eHAV virion is uncertain. It seems likely that both forms of the virus may interact with the same cellular receptor molecule, but in different cellular compartments: late endosomes/lysosomes after degradation of the membranes surrounding eHAV versus early endosomes or even the plasma membrane for HAV. However, this is conjecture. Subtle differences in capsid protein composition, with VP1 possessing an 8-kDa carboxy-terminal extension (pX) in eHAV that is not present in naked HAV (1), would be consistent with the use of distinct cellular receptors.

More than 20 years ago, Kaplan et al. (9) reported that TIM1 (T cell immunoglobulin and mucin-containing domain protein 1) (otherwise known as hepatitis A virus cellular receptor 1 [HAVCR1]) was an essential cellular receptor for HAV in African green monkey kidney cells. TIM1 belongs to a family of immunoglobulin-like domain-containing transmembrane proteins that include three members in humans (human TIM1, TIM3, and TIM4) and eight members in mice (murine TIM1 to TIM8), of which the human TIM1, TIM3, and TIM4 are direct orthologs of murine TIM1, TIM3, and TIM4, respectively (10). These proteins are expressed on the cell surface, with their N-terminal immunoglobulin-like (IgV) and mucin domains present in the extracellular milieu and their C-terminal sequences in the cytoplasm. An important feature of all TIM proteins is a highly conserved phosphatidylserine (PtdSer)-binding pocket in the IgV domain that recognizes PtdSer on the outer membrane leaflet of apoptotic cells, facilitating their uptake by phagocytic cells (11). Importantly, TIM1 and TIM4 have also been shown to facilitate attachment of a variety of enveloped viruses, including filoviruses, alphaviruses, and flaviviruses, that display PtdSer on their surface (12–14). Although TIM1 undergoes tyrosine phosphorylation of its C-terminal cytoplasmic tail, this is not required for facilitation of enveloped virus entry (14, 15).

The identification of TIM1 as a putative HAV receptor (9, 16) predates the more recent discovery of eHAV (1). The uptake of eHAV by plasmacytoid dendritic cells is partially blocked by recombinant annexin V (17), suggesting that eHAV (like exosomes and conventional enveloped viruses) displays PtdSer on its surface and that TIM1 might facilitate attachment and subsequent entry of eHAV, but not naked HAV, into cells by binding PtdSer on the eHAV surface. These considerations prompted us to revisit the role of TIM1 in hepatovirus entry. Here, we report the results of experiments that assess whether TIM1 is required for attachment and/or infection of permissive cell cultures or *Ifnar1*^*−/−*^ mice by either eHAV or HAV. While we show that TIM1 modestly promotes infection of Vero cells by eHAV, it is not an essential entry factor for either form of the virus.

## RESULTS

### TIM1 is not essential for eHAV or HAV infection of human hepatoma-derived cells.

**FIG 1:**
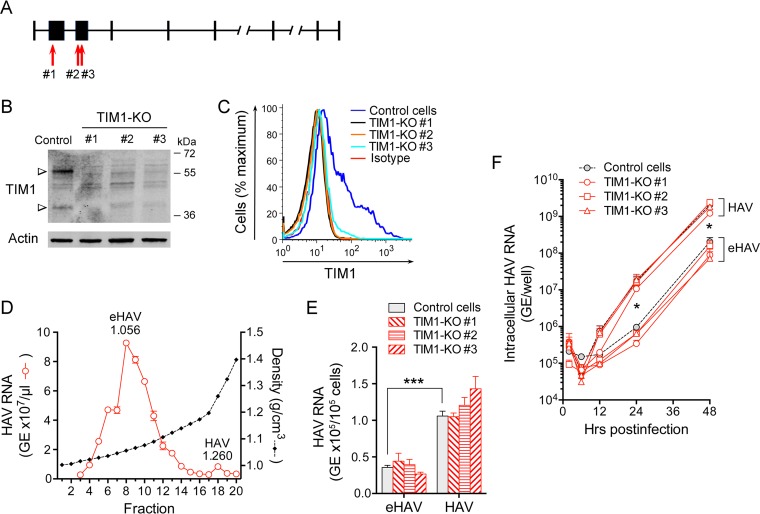
Impact of TIM1 knockout on eHAV and HAV infection of human Huh-7.5 cells. (A) Human HAVCR1 gene structure (NCBI *Homo sapiens* annotation release 108, accession no. XM_017009340.1; map not drawn to scale). The red arrows show the locations of CRISPR-induced disruption of the TIM1 sequence in exons 2 (KO#1) and 3 (KO#2 and KO#3). (B) Immunoblots of TIM1 and actin (loading control) in lysates of control Huh-7.5 cells and CRISPR/Cas9-generated Huh-7.5 TIM1-KO cells. Anti-TIM1 reactive bands appear at ∼38.7 kDa (predicted TIM1 molecular mass) and ∼55 kDa. (C) Surface expression of TIM1 on control Huh-7.5 and TIM1-KO cells quantified by flow cytometry. “Isotype” refers to the immunoglobulin control. (D) Distribution of HAV RNA in an isopycnic iodixanol density gradient loaded with supernatant fluids of HM175/18f-infected Huh-7.5 cells. The abundance of eHAV, the predominant form of virus present in supernatant fluids, peaked in fraction 8 (1.056 g/cm^3^), whereas naked HAV formed a small peak in fraction 18 (1.260 g/cm^3^). (E) Adherence of HAV and eHAV to control Huh-7.5 and Huh-7.5 TIM1-KO cells at 4°C determined by RT-qPCR specific for viral RNA. Differences in residual virus bound to parental versus TIM1-KO cells did not achieve statistical significance. Error bars = SEM; *n* = 6 (2 independent experiments, each with 3 technical replicates). (F) Accumulation of intracellular HAV RNA in Huh-7.5 and related TIM1-KO cells following infection at 37°C with eHAV or HAV inocula containing similar amounts of HAV RNA. Viral RNA in HAV-infected cells significantly exceeded that in eHAV-infected cells at 24 and 48 h, but differences between control and any TIM1-KO cell line infected with the same inoculum did not achieve statistical significance. Error bars = SEM; *n* = 4 (2 independent experiments each with 2 technical replicates). Values in panels E and F that were significantly different for eHAV versus HAV by two-way analysis of variance (ANOVA) are indicated by bars and asterisks as follows: *, *P* < 0.05; ***, *P* < 0.001.

Although TIM1 has been widely accepted to function as a cellular receptor for HAV based on early studies of African green monkey kidney cells (9), there is relatively little expression of TIM1 within the human liver (16). To assess its role in the cellular entry of naked HAV virions and quasi-enveloped eHAV into hepatocytes, we used lentivirus-mediated CRISPR/Cas9 gene editing to knock out (KO) TIM1 expression in Huh-7.5 cells. Huh-7.5 cells are derived from a human hepatoma and robustly support replication of the virus due to a lack of interferon induction pathways (18). PCR sequencing confirmed the locations of CRISPR-induced indels in genomic DNA from each of three independent puromycin-selected cell lines generated with different single guide RNAs (sgRNAs) targeting exons 2 and 3 (Fig. 1A). The protein products expressed from these cell lines are predicted to include only the first 31 (TIM1-KO#1), 141 (TIM1-KO#2), or 158 (TIM1-KO#3) N-terminal amino acids of TIM1, and thus lack the C-terminal TIM1 membrane anchor. Immunoblotting and flow cytometry assays confirmed >95% reduction in TIM1 protein expression in each knockout cell line (Fig. 1B and C). To ascertain whether there are differences in the attachment of eHAV or HAV virions to these cells, we incubated the cells at 4°C with dilutions of fractions from an isopycnic gradient (Fig. 1D) containing equal quantities of eHAV or naked HAV (based on HAV RNA content) produced in Huh-7.5 cells. The inocula were removed after 2 h, the cells were washed extensively, and virus remaining bound to the cells was quantified by quantitative reverse transcription-PCR (RT-qPCR) (Fig. 1E). Overall, less quasi-enveloped eHAV remained bound to the cells compared to naked HAV, indicating that eHAV attachment to Huh-7.5 cells is less efficient than HAV attachment (*P* < 0.001). Similar observations have been reported for eHEV versus HEV attachment (19). However, the absence of TIM1 expression did not result in any difference in the adherence of either eHAV or HAV.

Next, we measured the accumulation of intracellular viral RNA over time following infection of the cells at 37°C, with the inocula removed and cells washed after a 1-h period of viral adsorption. The estimated multiplicity of infection (MOI) in these experiments was approximately 0.025 based on HAV RNA quantitation (10 genome equivalents [GE] per cell) and a specific infectivity of ∼400 GE/infectious unit (1). The quantity of HAV RNA detected in each of the three TIM1-KO cells was somewhat less than that in control cells between 6 and 48 h after infection with quasi-enveloped eHAV, but these differences did not achieve statistical significance (Fig. 1F). Smaller differences were evident in the cells infected with naked HAV. Interestingly, there was an initial delay of about 12 h in the replication of eHAV compared to HAV in both control and TIM1-KO cells (Fig. 1F). This is likely to reflect the slow endolysosomal entry route taken by eHAV, in contrast to the relatively rapid entry of naked HAV (1). Intracellular viral RNA increased subsequently at similar rates in cells infected with either eHAV or HAV after the first 24 h of infection. This reflects the fact that the virus released from cells after the first round of replication is quasi-enveloped, regardless of whether the inoculum was HAV or eHAV. Collectively, these data indicate that TIM1 is not an essential entry factor for either eHAV or HAV in human hepatoma cells.

### TIM1 is not required for eHAV or HAV infection of Vero cells.

**FIG 2:**
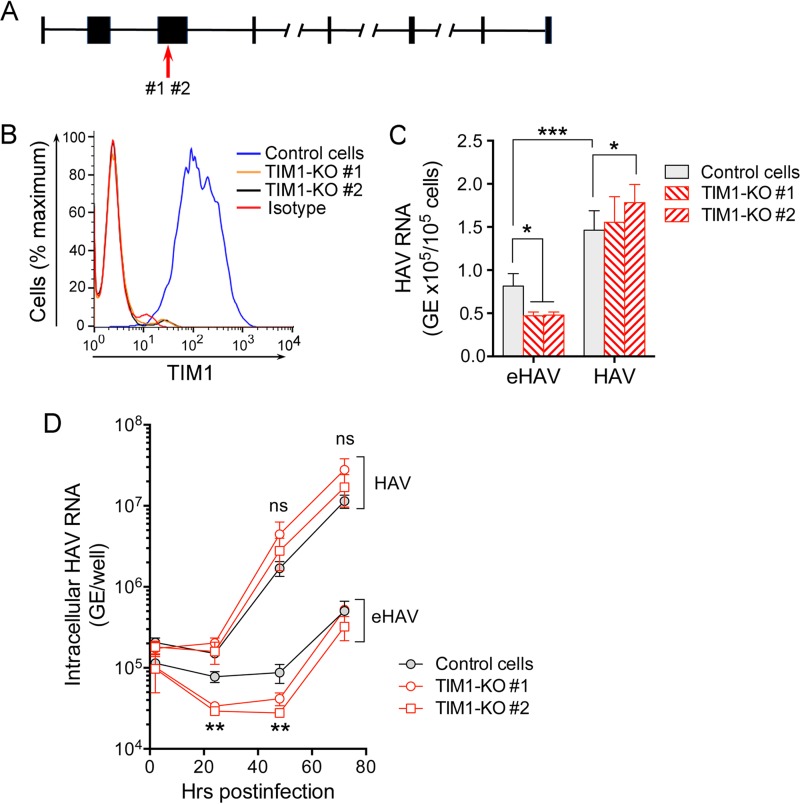
Impact of TIM1 knockout on HAV infection of Vero cells. (A) African green monkey HAVCR1 gene structure (NCBI *Chlorocebus sabaeus* annotation release 100, accession no. XM_008015132.1; map not drawn to scale). The red arrow shows the location of CRISPR-induced disruption of the TIM1 sequence in exon 3 as determined by DNA sequencing. (B) Expression of TIM1 on the surfaces of control and TIM1-KO Vero cells quantified by flow cytometry. “Isotype” refers to the immunoglobulin control. (C) Adherence of HAV and eHAV to Vero control and two distinct TIM1-KO cell lines at 4°C, determined by RT-qPCR specific for viral RNA. Error bars = SEM; *n* = 5 (2 independent experiments, each with 2-3 technical replicates). *, *P* < 0.05; ***, *P* < 0.001. (D) Accumulation of intracellular HAV RNA in control and TIM1-KO Vero cells following infection at 37°C with eHAV or HAV inocula containing similar amounts of HAV RNA. Viral RNA in quasi-enveloped eHAV-infected control cells exceeded that in TIM1-KO cells at 24 and 48 h (TIM1-KO#1 [*P* < 0.05] and TIM1-KO#2 [*P* < 0.01] by two-way ANOVA with Tukey’s multiple-comparison test). Error bars = SEM; *n* = 4 (2 independent experiments each with 2 technical replicates). There was no significant difference (ns) between HAV RNA levels in control and KO cell lines infected with naked HAV.

TIM1 (originally named HAVCR-1) was first reported to be a receptor for HAV in cells from an African green monkey (*Clorocebus aethiops*) (9). Since human TIM1 shares only ∼79% amino acid identity with the *C. aethiops* ortholog, we also assessed HAV attachment and replication in Vero cells that are derived from *Clorocebus sabaeus* (20). TIM1 is predicted to be 99% identical in these two African green monkey species. Using the same inocula as those in Fig. 1, we first measured attachment of virus to two distinct clones of Vero TIM1-KO cells generated by CRISPR/Cas9 using sgRNAs targeting exon 3. Sequencing of genomic DNA demonstrated that both clones express C-terminally truncated TIM1, containing only the N-terminal 133 amino acids and lacking the C-terminal TIM1 membrane anchor (Fig. 2A). Both clones demonstrated a complete loss of surface expression of TIM1 (Fig. 2B). As with Huh-7.5 cells (Fig. 1E), eHAV adhered less to Vero cells than naked HAV did (*P* < 0.001) (Fig. 2C). However, in contrast to what we observed with Huh-7.5 TIM1-KO cells, eHAV attachment at 4°C was reduced about twofold in each of the two Vero TIM1-KO cell lines compared to Vero control cells (*P* < 0.05 by two-way analysis of variance [ANOVA]). Naked HAV virions showed no reduction but rather a slight increase in adherence to these KO cells (*P* < 0.05) (Fig. 2C). These results can be explained by the PtdSer-binding activity of TIM1, since previous studies show PtdSer is displayed on the surfaces of eHAV virions (17). TIM1 is expressed at much higher levels in the kidney than in the liver (16), and a greater density of TIM1 expressed on the surfaces of Vero cells that are derived from kidney cells may explain why its loss has more effect in these cells than in Huh-7.5 cells that are derived from the liver. However, species-specific differences in sequence and antigenicity of TIM1 make it difficult to directly compare the levels of expression in these two cell lines.

Next, we examined the replication kinetics of both forms of the virus in Vero TIM1-KO cells. As in Huh-7.5 cells (Fig. 1F), eHAV replication was significantly delayed compared to replication of the naked HAV inoculum, with less intracellular RNA detected between 24 and 48 h postinfection than at 2 h postinfection in cells infected with quasi-enveloped virus (Fig. 2D). Significantly less viral RNA was detected during this period of time in eHAV-infected TIM1-KO cells compared to Vero control cells (*P* < 0.01), suggesting a role for TIM1 in eHAV entry. Viral RNA levels subsequently increased and were similar in both KO and control cells by 72 h postinfection. The lengthy replication delay in eHAV-infected cells did not occur with the naked HAV inoculum. The levels of intracellular RNA did not differ significantly between control and TIM1-KO cells infected with naked HAV, although it was consistently slightly greater in the KO cells at 48 and 72 h postinfection. This may reflect somewhat greater adherence of HAV to these cells, as suggested by the results shown in Fig. 2C. Overall, the replication efficiency of both types of virus in Vero cells was less than in Huh-7.5 cells, both in kinetics and titers reached by 48 h postinfection. This could reflect either species-specific differences in host factors or possibly the presence of active RIG-I signaling in Vero cells, but not Huh-7.5 cells (18). Taken collectively, these data indicate that TIM1 may facilitate attachment and entry of quasi-enveloped eHAV into Vero cells but that TIM1 is not an essential receptor for the virus in these cells.

### Hepatitis A infection in TIM1- and TIM4-deficient mice.

**FIG 3:**
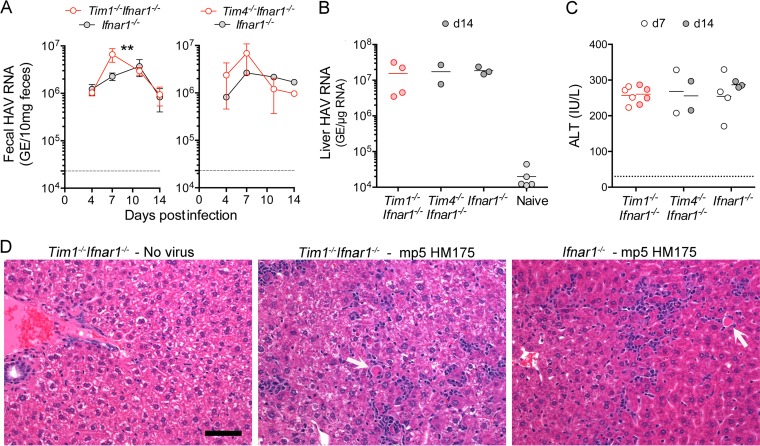
Hepatitis A infection in *Tim1*^*−/−*^* Ifnar1*^*−/−*^ (*n* = 4), *Tim4*^*−/−*^* Ifnar1*^*−/−*^ (*n* = 2), and *Ifnar1*^*−/−*^ (*n* = 4) mice inoculated intravenously (i.v.) with ∼10^8^ genome equivalents (GE) of fifth mouse-passage HM175 virus (mp5 unfractionated liver homogenate). (A) Fecal shedding of HAV by *Tim1*^*−/−*^* Ifnar1*^*−/−*^and *Tim4*^*−/−*^* Ifnar1*^*−/−*^double-knockout mice was similar to single *Ifnar1*^*−/−*^ knockouts, but significantly elevated in *Tim1*^*−/−*^* Ifnar1*^*−/−*^ versus *Ifnar1*^*−/−*^ mice on day 7 postinfection (*P* < 0.01). (B) Intrahepatic HAV RNA abundance at day 14 (d14) postinfection. (C) Serum ALT on days 7 and 14 postinfection. Bar = mean. (D) H&E-stained liver sections from mock-infected *Tim1*^*−/−*^* Ifnar1*^*−/−*^ mice, mp5 HM175 virus-infected *Tim1*^*−/−*^* Ifnar1*^*−/−*^ mice, and *Ifnar1*^*−/−*^ mice. Virus-infected *Tim1*^*−/−*^* Ifnar1*^*−/−*^ and *Ifnar1*^*−/−*^ livers show diffuse small inflammatory cell infiltrates with apoptotic hepatocytes (white arrows) scattered throughout the parenchyma. Only minimal numbers of periportal lymphocytes are evident in mock-infected liver tissue. Bar, 100 µm.

Our recent studies established a small animal model for HAV infection using *Ifnar1*^−/−^ mice (2). Wild-type HAV is highly hepatotropic in these animals, inducing features of acute hepatitis A that are typical in humans, including high levels of replication in the liver, but not intestinal tissue, coupled with fecal shedding of virus secreted from the liver via the biliary track (2, 3). Liver injury in the acute phase of the infection in *Ifnar1*^*−/−*^ mice is marked by elevation of serum alanine aminotransferase (ALT) activity and due to mitochondrial antiviral signaling protein (MAVS)- and interferon regulatory factor 3 (IRF3)/IRF7-dependent, but interferon-independent apoptotic death of infected hepatocytes. To determine whether the murine ortholog of TIM1 plays a role in hepatovirus pathogenesis in this animal model, we compared infections in *Tim1*^−/−^ *Ifnar1*^−/−^ versus single-knockout *Ifnar1*^−/−^ mice inoculated intravenously with fifth murine passage HM175 virus recovered from the liver of an infected *Mavs*^*−/−*^ mouse (Fig. 3). Fecal virus shedding was comparable in *Tim1*^−/−^ *Ifnar1*^−/− ^mice and *Ifnar1*^−/−^ mice, except at day 7 postinfection when it was greater in the *Tim1*^−/−^ *Ifnar1*^−/−^ double knockout (Fig. 3A). There were no differences in intrahepatic HAV RNA (Fig. 3B), serum ALT elevation (Fig. 3C), or histopathology of the liver at necropsy on day 14 (Fig. 3D). Infected liver tissues from both *Tim1*^−/−^ *Ifnar1*^−/−^ and *Ifnar1*^−/−^ mice contained similar numbers of apoptotic hepatocytes and comparable inflammatory cell infiltrates.

TIM4 is closely related to TIM1 and well conserved in humans and mice (10). TIM4 has a longer mucin domain than TIM1, and it has a PtdSer-binding pocket as well as a putative integrin-binding site in its Ig-like domain (21, 22). Whereas TIM1 is expressed on T cells, TIM4 is expressed on the surfaces of antigen-presenting cells and contributes to the clearance of apoptotic bodies (22, 23). As with genetic deletion of *Tim1* in *Ifnar1*^*−/−*^ mice, *Tim4* deletion had no apparent effect on viral replication or liver pathogenesis in *Tim4*^*−/−*^* Ifnar1*^*−/−*^ mice infected with the liver-derived inoculum (Fig. 3A, B, and C). Taken together, these data indicate that neither TIM1 nor TIM4 is essential for hepatitis A replication or pathogenesis in *Ifnar1*^*−/−*^ mice.

### Replication kinetics and liver injury in eHAV-infected versus HAV-infected *Tim1*^−/−^ *Ifnar1*^−/−^ mice.

**FIG 4:**
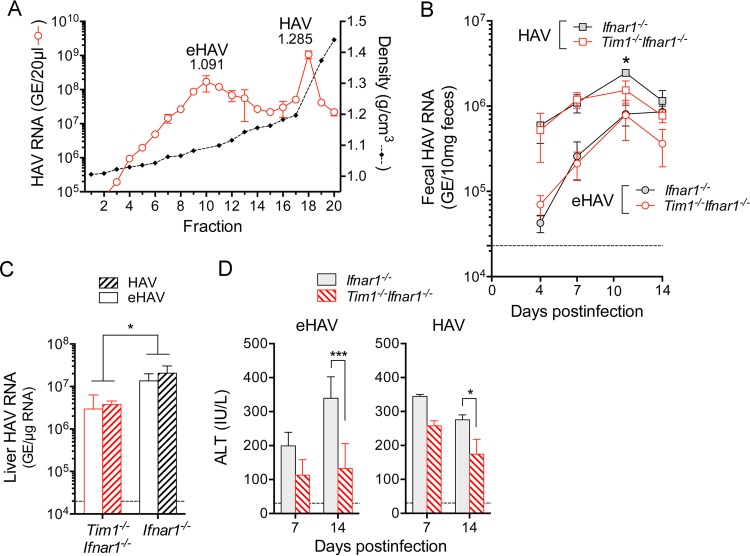
Infection in *Ifnar1*^*−/−*^ and *Tim1*^*−/−*^* Ifnar1*^*−/−*^ mice (four mice in each group) initiated by gradient-isolated quasi-enveloped and naked, nonenveloped fifth mouse-passage virus. (A) HAV RNA in fractions of an isopycnic iodixanol gradient loaded with a lysate of infected *Mavs*^*−/−*^ mouse liver (2). Fractions 10 (eHAV) and 18 (HAV) were used to inoculate mice with comparable amounts of virus based on RNA quantitation (∼10^8^ genome equivalents). (B) Fecal HAV shedding in mice infected by i.v. inoculation of HAV or eHAV (fractionated as described for panel A). Fecal shedding was significantly greater in HAV-infected than eHAV-infected animals at 7 and 10 days postinfection, regardless of the presence or absence of TIM1 (*P* < 0.001 by two-way ANOVA). *Ifnar1*^*−/−*^ and *Tim1*^*−/−*^* Ifnar1*^*−/−*^ mice differed in fecal shedding only at day 10 in HAV-infected, not eHAV-infected animals (*P* < 0.05). There were four mice in each group. (C) Intrahepatic HAV RNA at day 14 postinfection. Less viral RNA was present in *Tim1*^*−/−*^* Ifnar1*^*−/−*^ mice than in *Ifnar1*^*−/−*^ mice infected with either inoculum (*P* < 0.05 by two-way ANOVA). (D) Serum ALT on days 7 and 14 postinfection. ALT elevations were consistently lower in *Tim1*^*−/−*^* Ifnar1*^*−/−*^ mice compared to *Ifnar1*^*−/−*^ mice, but this difference achieved statistical significance only at day 14. Error bars = SEM. *, *P* < 0.05; ***, *P* < 0.001.

Cell culture-adapted virus does not replicate efficiently in *Ifnar1*^*−/−*^ mice (data not shown), which is consistent with the previously reported loss of replication capacity of cell culture-adapted virus in primates (24). The titer of membrane-associated virus circulating in the blood of infected *Ifnar1*^*−/−*^ or *Mavs*^−/−^ mice is also insufficient to initiate infection in mice (2). Thus, to compare infections initiated by eHAV and HAV in *Tim1*^−/−^ *Ifnar1*^−/−^ mice, we prepared inocula by iodixanol gradient separation of naked, nonenveloped virions and membrane-associated virus present in a homogenate of infected *Mavs*^*−/−*^ mouse liver. Unlike virus recovered from supernatant fluids from infected cell cultures (Fig. 1D), the majority of virions present in infected liver homogenate are nonenveloped (HAV, fraction 18, 1.285 g/cm^3^) (Fig. 4A). Presumably, these naked virus particles are mostly intracellular in origin. However, a large amount of infectious membrane-cloaked virus with density indistinguishable from the density of extracellular eHAV is also present in liver lysate (eHAV, fraction 10, 1.091 g/cm^3^) (Fig. 4A). We infected *Tim1*^−/−^ *Ifnar1*^−/−^ and *Ifnar1*^−/−^ mice by intravenous inoculation of dilutions of these fractions containing equivalent HAV genome copy numbers. Fecal shedding was slower in onset in both types of mice following challenge with either inoculum than in mice infected with unfractionated virus (compare Fig. 4B and 3A), suggesting that the gradient-purified eHAV and HAV inocula possessed lower infectious titers than the unfractionated homogenate did, despite comparable amounts of viral RNA. However, with the exception of a small but statistically significant difference at day 11 in HAV-infected mice, there was no difference in fecal shedding by *Tim1*^−/−^ *Ifnar1*^−/−^ versus *Ifnar1*^−/−^ mice infected with the same inoculum (Fig. 4B). Consistent with the *in vitro* studies described above, the naked HAV inoculum was considerably more infectious than the membrane-associated eHAV inoculum, leading to higher initial fecal virus shedding on day 4 postinfection (Fig. 4B). Despite this, there was no significant difference in the amount of viral RNA in the livers of eHAV- versus HAV-infected animals at 14 days (Fig. 4C), likely because viral spread beyond the first round of replication is due to eHAV in both cases (2, 3). Importantly, however, regardless of the inoculum type, there were greater numbers of HAV genomes in the livers of *Ifnar1*^−/−^ mice compared to *Tim1*^−/−^ *Ifnar1*^−/−^ double-knockout mice (*P* < 0.05) (Fig. 4C), indicating that TIM1, while not required as a cellular receptor, does promote replication and probably spread of the virus *in vivo*.

**FIG 5:**
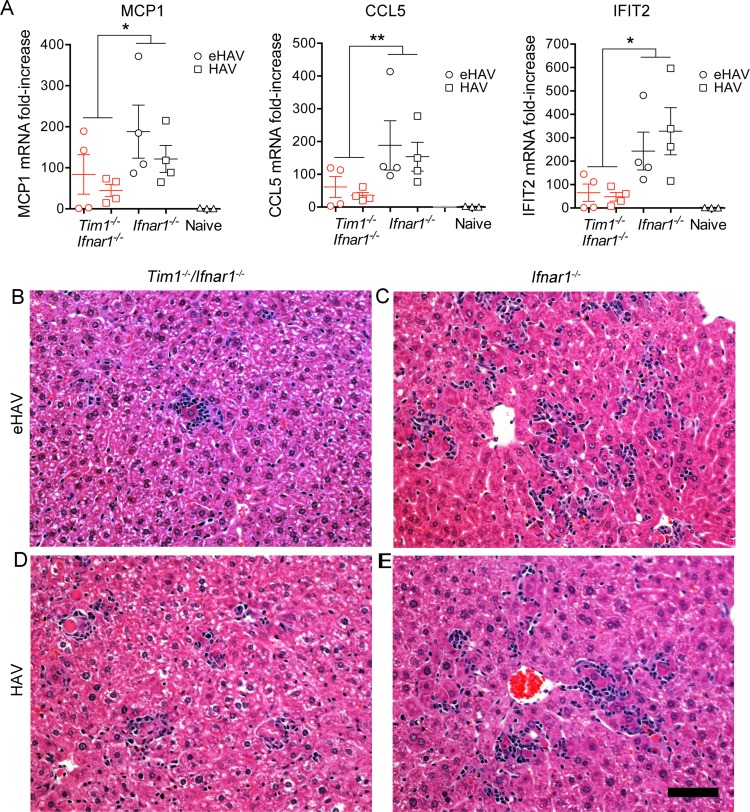
Interferon-stimulated gene expression and histopathology in the livers of eHAV- or HAV-infected *Tim1*^*−/−*^* Ifnar1*^*−/−*^ mice versus *Ifnar1*^*−/−*^ mice, 14 days postinfection. (A) Intrahepatic MCP-1, CCL5 (Rantes), and IFIT-2 mRNA expression. Data shown represent the mean ± SEM fold increase compared to uninfected control mice. There were four mice in each group. *, *P* < 0.05 by two-way ANOVA; **, *P* < 0.01 by two-way ANOVA. (B, C) H&E-stained liver sections from eHAV-infected mice: (B) *Tim1*^*−/−*^* Ifnar1*^*−/−*^ double-knockout (ALT = 249 IU/ml) showing a single focus of inflammation, characterized by an apoptotic hepatocyte and a surrounding aggregate of infiltrating lymphocytes; (C) *Ifnar1*^*−/−*^ single-knockout (ALT = 309 IU/ml). (D, E) Similar sections from naked HAV virion-infected (D) *Tim1*^*−/−*^* Ifnar1*^*−/−*^ (ALT = 290 IU/ml) and (E) *Ifnar1*^−/−^ (ALT = 270 IU/ml) mice, each showing a diffuse inflammatory infiltrate of lymphocytes and scattered apoptotic hepatocytes. Bar, 100 µm.

Consistent with lower levels of intrahepatic viral RNA in both eHAV- and HAV-infected *Tim1*^−/−^ *Ifnar1*^−/−^ versus *Ifnar1*^−/−^ animals, serum ALT activities were also lower in *Tim1*^−/−^ *Ifnar1*^−/−^ mice 7 and 14 days after infection (Fig. 4D). Liver injury in HAV-infected *Ifnar1*^−/−^ mice occurs independently of T cell or NK cell responses to HAV infection and likely results from IRF3-dependent induction of proapoptotic interferon-stimulated genes (ISGs) (2). We monitored ISG induction in infected *Tim1*^−/−^ *Ifnar1*^−/−^ double-knockout and *Ifnar1*^−/−^ single-knockout mice by quantifying intrahepatic MCP1 (macrophage chemoattractant 1), CCL5 (CC chemokine ligand 2) (Rantes), and IFIT2 (interferon-induced protein with tetratricopeptide repeats 2) mRNA transcript levels 14 days postinfection. All three of these ISGs were significantly less induced in infected *Tim1*^−/−^ *Ifnar1*^−/−^ mice compared to *Ifnar1*^−/−^ mice (Fig. 5A). While variable, histopathologic changes in the liver were also less impressive in *Tim1*^−/−^ *Ifnar1*^−/−^ mice than in *Ifnar1*^−/−^ mice, although all infected animals demonstrated inflammatory infiltrates in association with apoptotic hepatocytes (Fig. 5B to E). Collectively, these data indicate that TIM1 is not essential for hepatovirus to infect and replicate in *Ifnar1*^*−/−*^ mice, although it may promote spread of the virus within the liver and attendant liver injury.

## DISCUSSION

Previous studies of poliovirus and other picornaviruses have shown that direct interactions between the viral capsid and a cellular receptor molecule are central to uncoating and delivery of the RNA genome across endosomal membranes to the cytosol (8, 25). Since its initial identification in 1996, TIM1 (HAVCR1) has been widely accepted to be the cellular receptor for HAV (9, 16). Despite considerable published evidence supporting a role for TIM1 in the cellular entry of HAV, we provide definitive evidence here in human, simian, and murine systems that TIM1 is not an essential host factor for HAV infection. TIM1 facilitates the adherence and subsequent entry of many conventionally enveloped viruses by binding PtdSer displayed on the viral envelope (21). Our data are consistent with a similar role for TIM1 in the entry of quasi-enveloped eHAV into monkey kidney cells. Recombinant annexin V inhibits the uptake of quasi-enveloped eHAV by plasmacytoid dendritic cells, indicating that PtdSer is displayed on the surface of the eHAV membrane (17), and adherence of eHAV (but not HAV) to Vero cells was significantly reduced by knocking out TIM1 expression (Fig. 2C). Early replication kinetics of quasi-enveloped eHAV, but not naked HAV, were similarly affected (Fig. 2D). However, CRISPR/Cas9-mediated knockout of TIM1 did not impair infection of either Huh-7.5 human hepatoma cells or Vero cells by naked HAV (Fig. 1F and 2D).

Compared to *Ifnar1*^*−/−*^ mice, *Tim1*^*−/−*^* Ifnar1*^*−/−*^ mice demonstrated less fecal HAV shedding (Fig. 4B), as well as a 0.5-log-unit difference in the abundance of viral RNA in the liver (Fig. 4C), smaller increases in ALT levels (Fig. 4D), and lower cytokine levels (Fig. 5A) 14 days after infection with gradient-purified eHAV or HAV. These modest differences suggest an accessory role for murine TIM1 in infection of *Ifnar1*^*−/−*^ mice, possibly related to PtdSer binding facilitating the spread of quasi-enveloped eHAV (produced by both quasi-enveloped eHAV and naked HAV inocula) within the liver. Such differences were not evident in mice infected with higher-titer, unfractionated liver homogenate for which secondary spread in the liver may have been less important (Fig. 3).

The identification of TIM1 as a receptor for HAV (9, 16) predated discovery of the quasi-envelopment of HAV as the mechanism responsible for nonlytic release of virus from infected hepatocytes (1). The nature of the viral inoculum used in the early studies of TIM1 was not described well enough to know whether it was predominantly eHAV or HAV. There is, for example, no mention of whether it was detergent treated or gradient purified or whether it was derived from cell lysate or cell culture supernatant fluids. It is possible that the PtdSer-binding activity of TIM1 may have confounded the interpretation of these studies if the inoculum contained a large proportion of quasi-enveloped virions. The impact of PtdSer binding by TIM1 could have been particularly prominent in the GL37 cells used in these studies, as they are derived from African green monkey kidney cells (9). TIM1 expression is particularly high in the kidney, and we noted a much greater impact of TIM1 knockout on eHAV binding in Vero cells (Fig. 2C) than in Huh-7.5 cells derived from a human hepatoma (Fig. 1E). Some early studies suggested that HAV (or eHAV?) infectivity could be neutralized by a recombinant soluble protein representing the extracellular domain of TIM1 (26). In retrospect, TIM1 may have exerted a neutralizing effect by binding PtdSer on the surfaces of quasi-enveloped virions in these experiments.

If TIM1 is not the cellular receptor for HAV, then what is? At present, we can only speculate. Given the close antigenic relatedness between the capsids of bat and human hepatoviruses and the ability of human HAV to readily infect mice with defects in innate immunity (2, 27), we suggest that the HAV receptor is likely to be a protein that is widely expressed in nature and conserved through evolution. Because HAV infects a variety of cultured cells derived not only from the liver but also the kidney and lungs (28), the receptor is unlikely to be expressed only in hepatocytes or to explain the strong hepatotropism demonstrated by HAV *in vivo* (2). Our previously published work indicates that eHAV entry occurs slowly and is inhibited by the lysosomal poison chloroquine, leading us to hypothesize that the eHAV membrane is degraded within late endosomes or lysosomes (1). Recent evidence suggests that a similar mechanism accounts for the loss of membrane from quasi-enveloped eHEV (19). If the quasi-enveloped eHAV capsid then interacts with the same receptor (after removal of its membrane) as naked HAV virions, the receptor would be expressed on both late endosome-lysosomal membranes and membranes of early endosomes (or possibly the plasma membrane), and would thus be a protein that traffics between these compartments. Interestingly, TIM1 traffics in just such a fashion (29), and although it is not an essential entry factor, it could contribute to the internalization of quasi-enveloped eHAV and its movement to lysosomes in Vero cells with high TIM1 expression (Fig. 2B).

However, two alternative hypotheses should be considered. First, it is possible that the quasi-enveloped capsid, which contains an 8-kDa extension on each of its 60 VP1 molecules (1), may differ structurally from the naked viral particle. Consistent with this, tandem YPX_3_L “late domains” present in VP2 that appear to interact with the ESCRT-associated protein ALIX during the process of quasi-envelopment are mostly buried below the surface of the X-ray structure of the naked HAV capsid (30, 31). If their capsid structures do differ significantly, HAV and eHAV may interact with different receptors. Alternatively, Wang et al. (31) have speculated that HAV may enter cells via a mechanism completely distinct from the mechanisms of other picornaviruses, perhaps even uncoating its genome after internalization since there is evidence that HAV undergoes transcytosis across epithelial cells (32). This hypothesis is suggested by the X-ray model of the HAV capsid that shows no “canyon” surrounding the five-fold axis of symmetry such as that present in enteroviruses and into which the enteroviral receptor fits and no obvious alternative site with which a receptor might dock (31). This seems an unlikely scenario, however, as HAV transcytosis is very inefficient in the absence of antibodies to the capsid (3, 33).

Numerous questions remain to be answered concerning the mechanisms of entry for both naked HAV and quasi-enveloped eHAV virions. However, the data presented here correct the record regarding the role played by TIM1 in hepatovirus entry and are certain to stimulate future efforts to find essential host factors required for hepatovirus entry.

## MATERIALS AND METHODS

### Cells.

Huh-7.5 cells (obtained from Charles Rice, Rockefeller University) and Vero cells were maintained in Dulbecco’s modified Eagle medium supplemented with 10% fetal bovine serum (FBS), 100 U/ml penicillin G, and 100 µg/ml streptomycin at 37°C in a 5% CO_2_ atmosphere.

### CRISPR/Cas9 knockout of TIM1.

To produce Huh-7.5 cells lacking expression of TIM1, CRISPR/Cas9-expressing lentiviruses were generated by transfection of 293T cells (∼2.5 × 10^5^) with 1 µg of TIM1-targeting or control lentivector plasmid DNA along with third-generation lentivirus packaging mix (Applied Biological Materials, Richmond, BC, Canada). At 48 to 72 h posttransfection, supernatant fluids were collected and passed through a 0.22-µm filter syringe. Aliquots were stored at −80°C until use. For lentiviral transduction, Huh-7.5 cells grown to 30% confluence (∼2.5 × 10^5^ cells) were overlaid with 1 ml of lentivirus supernatant supplemented with 8 µg/ml Polybrene (Sigma) and incubated overnight. TIM1-KO#1 cells were transduced with vector expressing the single guide RNA (sgRNA) TGGCAGGGTAGTGTGACAGA (TIM1 exon 2), TIM1-KO#2 cells were transduced with vector expressing sgRNA GCTCGTTCGAACAGTCGTGA (exon 3), and TIM1-KO#3 cells were transduced with vector expressing sgRNA GTTGTTGGAACAGTTGTCGT (exon 3) (catalog no. K0931005; Applied Biological Materials). Control cells were transduced with lentiviral vector expressing a scrambled, nontargeting sgRNA, GCACTCACATCGCTACATCA (catalog no. KO-10; Applied Biological Materials). On the following day, 1 ml of complete medium was added, and the cells were incubated for an additional 48 h. The cells were washed with 1× phosphate-buffered saline (PBS) and fresh complete media supplemented with 6 µg/ml puromycin (InvivoGen) for 24 h. Dead cells were removed, and puromycin selection was continued with a change of medium every 3 or 4 days until the cells were fully confluent. The location of CRISPR-induced mutations in knockout cells was confirmed by sequencing PCR amplimers from genomic DNA.

To produce Vero TIM1 knockout cell lines, CRISPR/Cas9 targets (GX_20_GG) were identified in genomic DNA within TIM1 exons, and sequences modified to include BbsI restriction sites on either end were synthesized as oligonucleotides along with their reverse complement. Complementary oligonucleotides were annealed and ligated with BbsI-digested pX330-U6-Chimeric_BB-CBh-hSpCas9 (pX330; a kind gift from Kimberly Leslie, University of Iowa). Cloned constructs were confirmed by Sanger sequencing. Knockout (KO) cell lines (Vero TIM1-KO#1 and Vero TIM1-KO#2) were produced by transfecting Vero cells with plasmids expressing Cas9 and a pair of sgRNAs targeting exon 3, GTTCGAACAGTTCTGACAAT and GTAGAAACCATGGTTGTCGT. Clonal populations were generated by single-cell cloning, and expanded populations that were selected as TIM1 KO lines were verified at the genomic level by PCR followed by DNA sequencing and at the expression level by cell surface staining and flow cytometry. Knockout cell lines were maintained under the same conditions as wild-type cell lines.

### Preparation of purified eHAV and HAV.

Cell culture supernatant fluids from HAV-infected Huh-7.5 cells (8 to 10 days postinfection) were centrifuged at 1,000 × *g* for 10 min at 4°C to remove debris and further clarified by a spin at 10,000 × *g* for 30 min. The virus was concentrated by ultracentrifugation at 100,000 × *g* for 1 h. The resulting pellet was resuspended in 250 µl PBS and then loaded on top of a five-step gradient of 8 to 40% iodixanol and centrifuged at 165,915 × *g* (37,000 rpm) in a Beckman SW55i rotor for 24 h at 4°C using a Beckman Optima LE-80K ultracentrifuge. Approximately 20 fractions were collected from the top, and HAV RNA content and density were quantified by reverse transcription-PCR (RT-qPCR) (Bio-Rad) and refractometry (Mettler Toledo 30GS), respectively. Fractions containing quasi-enveloped and naked viruses at the appropriate buoyant densities (for eHAV, approximately 1.08 g/cm^3^, fractions 8 to 10; for nonenveloped HAV, approximately 1.22 g/cm^3^, fraction 18) were stored in aliquots at −80°C until use.

### Viral adherence and infection assay assays.

For virus attachment assays, 2 × 10^6^ GE (genome equivalents) of eHAV and HAV were incubated with 1 × 10^5^ control or TIM1-KO cells at 4°C for 2 h. Following two washes with 1× PBS, the cells were lysed in 350-µl RLT buffer and total RNA was extracted using the RNeasy kit (Qiagen, Germany). The number of bound viral genomes was quantified by two-step TaqMan-based quantitative RT-PCR (RT-qPCR). Briefly, total RNA was eluted in 35 µl diethyl pyrocarbonate (DEPC)-treated water, and a 4-µl aliquot of this water was used in a 10 µl cDNA synthesis reaction mixture with an oligo(dT) primer. cDNA synthesis was carried out with SuperScript III first-strand synthesis supermix for RT-qPCR kit (Invitrogen). The cDNA was diluted twofold, and a 2-µl aliquot was used in each well of a 96-well plate for a RT-qPCR. HAV RNA levels were determined by reference to a standard curve generated with synthetic HAV RNA. Primers targeted sequences in the 5′ untranslated RNA segment of the genome: 5′ GGTAGGCTACGGGTGAAAC 3′ and 5′-AACAACTCACCAATATCCGC 3′ (2). The 6-carboxyfluorescein (FAM)/6-carboxytetramethylrhodamine (TAMRA) probe was 5′ CTTAGGCTAATACTTCTATGAAGAGATGC 3′. Amplifications were carried out with 2× TaqMan universal PCR master mix (Life Technologies).

For viral replication assays, 2 × 10^6^ GE of eHAV and HAV were inoculated onto 1 × 10^5^ control or TIM1 knockout cells and incubated at 37°C for 1 h. The inoculum was then removed, and the cells were washed and reincubated at 37°C. Culture supernatant fluids were removed, and the cells were washed twice with 1× PBS and subsequently lysed at intervals. Viral RNA was quantified as described above.

### Mice.

*Ifnar1*^−/−^ mice, originally produced by U. Muller (34), were backcrossed with C57BL/6 mice for more than 10 generations and bred at the University of North Carolina at Chapel Hill (2). *Tim1*^*−/−*^* Ifnar*^*−/−*^ and *Tim4*^*−/−*^* Ifnar*^*−/−*^ mice were generated at the University of Iowa by crossing C57BL/6 *Tim1*^*−/−*^ and C57BL/6 *Tim4*^*−/−*^ mice independently with C57BL/6 *Ifnar1*^*−/−*^ mice. Heterozygous progeny were interbred, and all expected genotypes were produced in normal Mendelian ratios. Genomic DNA from mouse tail clips was assessed by PCR to determine genotypes. The primers and protocol for *Ifnar1*^*−/−*^ screening have been previously described (35). *Tim1* primer sequences included the following: shared forward, 5′ GTTTGCTGCCTTATTTGTGTCTGG 3′; wild-type reverse, 5′ CAGACATCAACTCTACAAGGTCCAAGAC 3′; knockout reverse, 5′ GTCTGTCCTAGCTTCCTCACTG 3′. *Tim4* genotyping primers have been previously described (23). PCR amplification with both *Tim* primer sets was performed for more than 30 cycles, with 1 cycle consisting of 30 s at 94°C, 30 s at 55°C, and 1 min at 72°C. All use of mice at the University of North Carolina at Chapel Hill and the University of Iowa was approved by the appropriate Institutional Animal Care and Use Committee (IACUC).

### HAV infectious challenge of mice.

Mice were infected at 6 to 10 weeks of age at the University of North Carolina at Chapel Hill by intravenous inoculation of a homogenate of liver from a *Mavs*^*−/−*^ mouse infected with HM175 virus (fifth murine passage, ∼10^8.4^ GE of HAV RNA) (2). To isolate eHAV and HAV virions from this material, the homogenate was clarified by centrifugation at 10,000 × *g* for 30 min. A 250-µl aliquot of the clarified suspension was loaded onto an 8 to 40% iodixanol gradient and centrifuged at 165,915 × *g* (37,000 rpm) for 24 h at 4°C. Fractions were collected and assayed for HAV RNA as described above and as shown in Fig. 4A and stored at −80°C until use. Infected mice were housed in individual cages for collection of fecal pellets with periodic collection of serum samples. Tissues were harvested at necropsy 14 days after inoculation and stored in RNAlater (Thermo Fisher Scientific, Maltham, MA) or fixed in 10% neutral phosphate-buffered formalin for 48 h and then stored in 70% ethanol until processed for histology.

### Alanine aminotransferase activity.

Serum alanine aminotransferase (ALT) activity was measured using the MaxDiscovery ALT color endpoint assay kit (Bioo Scientific, Austin, TX).

### Quantitation of fecal virus shedding and intrahepatic HAV RNA.

RNA was extracted from fecal samples using the QiaAmp viral RNA isolation kit (Qiagen, Valencia, CA) (6). RNA was isolated from liver using TRIzol reagent (Invitrogen Life Technologies, Carlsbad, CA) according to the manufacturer’s suggested protocol. RNA concentration was measured using a NanoDrop instrument (Thermo Scientific, Wilmington, DE). HAV RNA was quantified in these samples by RT-qPCR using iScript one-step RT-qPCR kit for probes and the iTaq universal probes one-step kit (Bio-Rad, Hercules, CA) with a CFX96 real-time PCR detection system (Bio-Rad). HAV RNA levels were determined by reference to a standard curve generated with synthetic HAV RNA. HAV-specific primers and the FAM/TAMRA probe were as described above.

For quantitation of intrahepatic ISG mRNA, residual DNA was removed from RNA samples using RNase-free DNase (Qiagen). cDNA synthesis was carried out with SuperScript III first-strand synthesis supermix for RT-qPCR kit (Invitrogen). Probe sets for *Ccl2* (Mm00441242_m1), *Ifit2* (Mm00492606_m1), and *Ccl5* (Mm01302427_m1) were from TaqMan gene expression assays (Thermo Fisher Scientific). Amplifications were conducted with 2× TaqMan universal PCR master mix.

### Histopathology.

Formalin-fixed paraffin-embedded (FFPE) livers were sectioned at 4-μm thickness for histopathology and stained with hematoxylin and eosin (H&E). Slides were examined for histological changes by an expert veterinary hepatopathologist who was blind to experimental conditions.

### Immunoblotting.

Cells were lysed in radioimmunoprecipitation assay (RIPA) buffer containing 1% Triton X-100 for 15 min on ice and then clarified by centrifugation at 13,000 × *g* for 15 min. Total protein was quantified by the Bradford method. Samples were subjected to SDS-PAGE and immunoblotting by standard methods. The blots were blocked with Odyssey blocking buffer (LI-COR Bioscience) and probed with primary antibodies to human TIM1 (hTIM1) (1:1,000; clone 219211, Mab1750; R&D Systems) and β-actin (1:10,000; clone AC-74; Sigma). Infrared conjugated secondary antibodies (LI-COR Bioscience) were used for development. Protein bands were visualized with an Odyssey infrared imaging system (LI-COR Bioscience).

### Flow cytometry.

A total of 5 × 10^5^ Huh-7.5 TIM1 KO and control cells in each well of a six-well plate were treated with 1× trypsin-EDTA (Gibco), washed twice with fluorescence-activated cell sorting (FACS) buffer (1× PBS, 0.2% sodium azide, and 1% FBS), and transferred to the wells of a 96-well U-bottom plate. The cells were incubated with allophycocyanin (APC)-conjugated anti-human TIM1 (clone 1D12; BioLegend) or an isotype control antibody (APC mouse IgG1, κ) (clone MOPC-21; BioLegend) at a 1:300 dilution in 50-µl FACS buffer for 1 h in dark on ice. Following three washes with FACS buffer, cells were subjected to flow cytometry using a FACSCalibur cytometer (BD Biosciences). Data were analyzed with FlowJo software (Tree Star). TIM1 expression by Vero knockout cells was monitored by flow cytometry after staining with goat polyclonal anti-human TIM1 (catalog no. AF1750; R&D Systems).
